# Dramatic recovery of steroid-refractory relapsed multiple sclerosis following Fingolimod discontinuation using selective immune adsorption

**DOI:** 10.1186/s12883-015-0377-2

**Published:** 2015-07-31

**Authors:** Roberto De Masi, Salvatore Accoto, Stefania Orlando, Vincenzo De Blasi, Sergio Pasca, Rocco Scarpello, Leo Spagnolo, Adele Idolo, Antonella De Donno

**Affiliations:** Laboratory of Neuroproteomics, Multiple Sclerosis Centre, “F. Ferrari” Hospital, 73042 Casarano, Lecce Italy; Multiple Sclerosis Centre, “F. Ferrari” Hospital, 73042 Casarano, Lecce Italy; Complex Operative Unit of Neurology-Stroke Unit, “F. Ferrari” Hospital, 73042 Casarano, Lecce Italy; Complex Operative Unit of Nephrology and Dialysis, “F. Ferrari” Hospital, 73042 Casarano, Lecce Italy; Division of Neuroradiology, “F. Ferrari” Hospital, 73042 Casarano, Lecce Italy; Department of Biological and Environmental Sciences and Technologies, Laboratory of Hygiene, University of the Salento, Lecce, Italy

**Keywords:** Multiple sclerosis, Selective immuno adsorption (SIA), Fingolimod, Steroid-refractory rebound, Intravenous 6-methyl prednisolone (IVMP)

## Abstract

**Background:**

Selective immune adsorption (SIA) is an emerging method for treating immune-mediated neurological diseases, given its superior safety profile compared to plasma exchange (PEX). However, the available literature concerning Multiple Sclerosis includes no cases of SIA applied to steroid-refractory rebound after Fingolimod discontinuation.

**Case presentation:**

Here we report the case of a 32-year-old woman suffering from multiple sclerosis treated with Fingolimod and admitted to a Multiple Sclerosis Centre after drug discontinuation due to the occurrence of lymphopenia.

During the few weeks preceding admission, the patient experienced progressive and severe neurological deterioration that did not respond to an initial cycle of pulsed high doses of intravenous 6-methyl prednisolone (IVMP). Given the ineffectiveness of a second cycle of IVMP, the patient was treated with plasma immunoadsorption, leading to dramatic functional recovery. The patient then started a neuro-rehabilitation program.

About one month after the final SIA procedure the patient started Natalizumab-based therapy, while maintaning a stable neurological condition.

We noted significant modification of C3/C4 complement components and total gamma globulin concentrations (IgG) during SIA.

**Conclusions:**

Our observations show that however serious, steroid-refractory neurological deterioration occurring after Fingolimod discontinuation in multiple sclerosis can be treated with selective immune-adsorption therapy which thus represents a good alternative in these cases.

It could be speculated that this clinical condition was associated with pattern II of demyelination, given the good response to a form of treatment that acts on autoantibodies. Thus, SIA represented an effective therapeutic strategy for this case of relapsed MS as steroid-resistent rebound post Fingolimod cessation.

## Background

Selective immune adsorption (SIA) is a column-based method for eliminating pathogenetically immune-relevant elements from plasma by using the binding properties of tryptophan. A tryptophan-immobilized polyvinyl alcohol gel column (IM-TR 350) semi-selectively adsorbs such autoantibodies and several fractions of the complement, sparing albumin and other plasma humoral components [1–3]. Unlike plasma exchange (PEX), this medical application does not entail any supplementation of plasma components and it demonstrates better safety, fewer side effects and comparable results [4, 5]. Several immune-mediated neurological pathologies benefit from this therapy, including Myasthenia Gravis [6], Guillain Barrè [7] and Fisher’s syndromes [8]. The literature on the use of SIA in Multiple Sclerosis (MS) is not extensive [9–12] and it rarely considers cases of steroid-refractory rebound after Fingolimod discontinuation. Fingolimod, used to reduce disability accumulation in MS, antagonises sphingosine-1-phosphate receptor (SP1R) and inhibits lymphocyte egression from the lymph nodes causing their count fall. One such case, the first to be treated by SIA, is reported here.

In 2000, Lucchinetti and co-workers proposed four pathological MS patterns, depending on the relative prevalence of inflammation, demyelination, oligodendrocytes and remyelination [13]. The type II pattern, expressing the main inflammation through antibodies/plasma cells and their complements, is thought to be specific to relapsing-remitting MS (RRMS) [13] and retrobulbar optic neuritis (RBON) [14]. The same humoral inflammatory elements have been shown to be adsorbed by the IM-TR 350 column and this is considered effective in the treatment of this restricted morbidity group. Also, some authors suggest that patients with pattern II pathology MS are more likely to respond favourably to plasma exchange than those with patterns I or III [15]. Moreover, in a recent work, Pedotti and co-workers showed that immunoadsorption was applied successfully to one RRMS patient not responding to high-doses of steroids. In addiction, antibodies were suggested to play a pathogenic role by passive transfer in the EAE (experimental autoimmune encephalomyelitis) model [16]. This study seeks to verify whether steroid-refractory rebound after Fingolimod discontinuation should also be counted in this group, together with RRMS and RBON.

## Case presentation

**Fig. 1:**
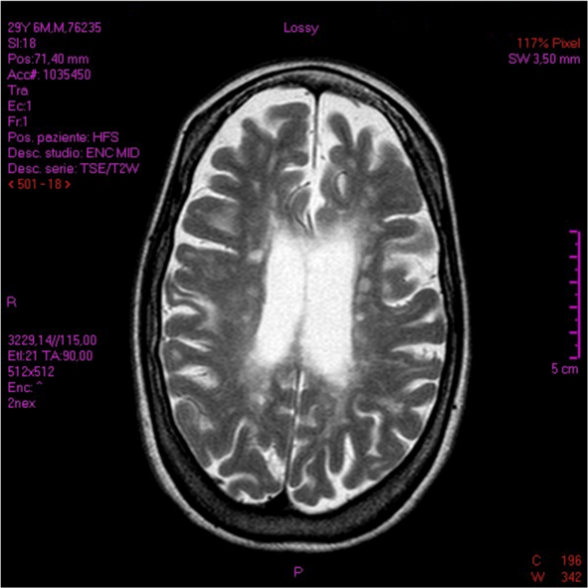
The brain MRI at baseline represented a moderate T2 lesion load

Here, we report the case of a 32-year-old woman affected by RRMS with disease onset at 17 years. After 10 years of therapy based on immunomodulators, the patient was treated with Fingolimod (orally 0.5 mg/day) for one and a half years preceding admission to hospital. This drug was indicated since the patient had relapsed with partial recovery, registering a worsening of 0.5 on the Expanded Disability Status Scale (EDSS) [17]. During this second line therapy, the EDSS was 5.0 and no side effects were signalled apart from the expected peripheral lymphopenia. Moreover, neurological evaluation showed only stable paraparesis with hyperreflexia in all limbs. In this period magnetic resonance imaging (MRI) scan evidenced a moderate lesion load, as shown in Fig. 1.

**Fig. 2:**
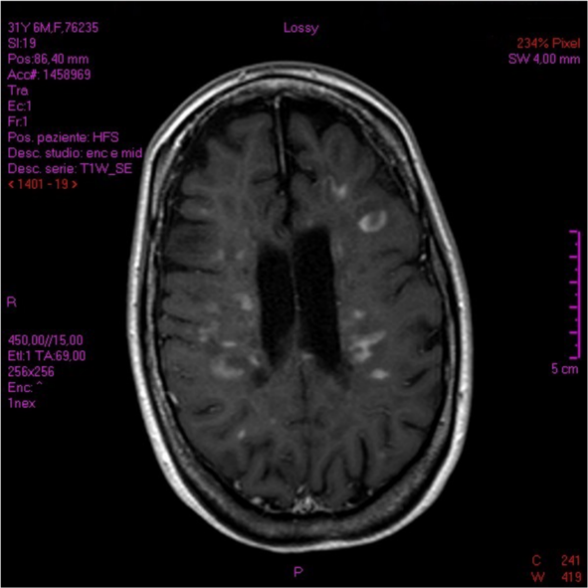
The pre-treatment brain MRI check-up demonstrated CELs. The main locations are supra- and infratentorial white matter, hemispheric white matter, cortical-subcortical junction, and periventricular white matter bilaterally

However, the lymphocytic count worsened in the weeks preceding admission (<200/μl) and Fingolimod was discontinued. About one month after this, the patient experienced sub-acute reduction in ambulatory autonomy (accumulation of disability with increasing paraparesis over 10 days) and underwent an initial cycle of intravenous 6-methyl prednisolone (IVMP) in pulsed high-doses (1 g/day for 5 days). Despite this, neurological condition rapidly worsened and the patient was admitted to the Multiple Sclerosis Centre at “F. Ferrari” Casarano Hospital. On the patient’s arrival in the Neurology Department, we detected subacute severe encephalopathy and multifocal neurological signs: serious spastic tetra-paresis, dysarthria, diplopia sustained by a divergent squint in OS, bowel and bladder disturbances and ideomotor slowdown with severe confusional state, amounting to an increase of 3.5 points in EDSS (8.5 total). No lymphopenia or fever was recorded and no infectious clinical/paraclinical signs were observed. MRI of the neuraxis demonstrated contrast-enhancing lesions (CELs) in the brain with ring enhancement, enlarging T2-lesions and an overall increase in lesion load with respect to the preceding radiological evaluation. The main locations were supra- and infra-tentorial white matter, hemispheric white matter, the cortical-subcortical junction, brainstem and periventricular white matter bilaterally (Fig. 2). Corticosteroid treatment and a neuro-rehabilitation program were rapidly instituted.

Two days after a second cycle of IVMP (as previously, 1 g/day for 5 days) administered without results, the patient was treated by SIA with echo-guided major venous access (via the right femoral vein, given the unsuccessful attempts via the right subclavian vein) in accordance with the following protocol: one session every 1 day for a total of five sessions. After 1 week, two further SIA sessions at a distance of 1 week from each other were planned. Each of which involving treatment blood volume of 2.6 litres in 2 h. Anticoagulation was obtained using intra venous sodium heparin at a first bolus dose of 250 UI, followed by 1000 UI/h for 2 h.

Unfortunately, after the fourth session, the patient had fever due to thrombophlebitis of the femoro-popliteal axis contiguous to the venous access and SIA was suspended. However, after the remission of the fever, she experienced dramatic neurological improvement with recovery of three points on the EDSS. Thus, in 3 weeks the patient reached EDSS 5.5 and maintained it using a monthly administration of Natalizumab as second-line therapy, instituted about a month after the final SIA procedure.

**Fig. 3:**
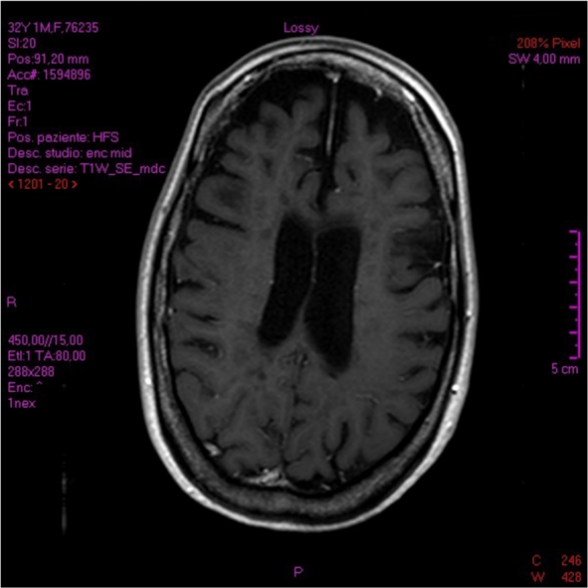
The post- treatment MRI check-up, performed 1 month after SIA, evidenced total regression of CELs

The post-treatment MRI, performed 1 month after SIA, evidenced total regression of CELs (Fig. 3) comparison respect to the pre-treatment MRI scan.

**Fig. 4:**
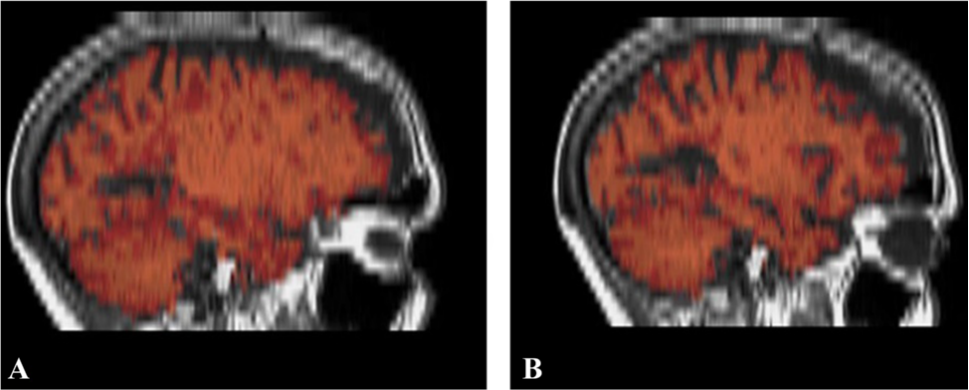
The MRI post-analyisis detected a reduction of 2 % in total brain volume (TBV) between before (**a**: TBV = 1246 ml) and after treatment (**b**: TBV = 1228 ml), as assessed by the Siena tool of FSL software

**Fig. 5:**
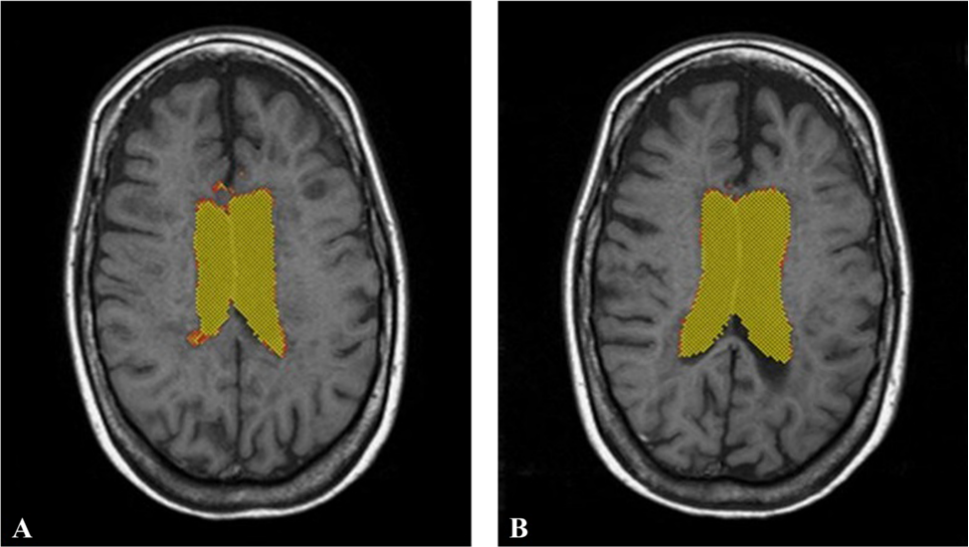
The MRI post-analyisis detected an increase of 17,5 % in cerebro-spinal fluid volume (CSFV) between before (**a**: CSFV = 94 ml) and after treatment (**b**: CSFV = 114 ml), as assessed by the Siena tool of FSL software

Moreover, MRI post-analysis detected substantial differences in total brain volume (TBV) and cerebrospinal fluid volume (CSFV). Pre-treatment values were TBV = 1246 ml and CSFV = 94 ml, while post-treatment values were TBV = 1228 ml and CSFV = 114 ml, as assessed by the FSL software Siena tool. Specifically, a reduction of 1.8 % in TBV and an increase of 17.5 % in CSFV were noted (Figs. 4 and 5).

From the clinical point of view, the patient improved her motor performance (“Pyr” EDSS sub-score from 4 to 3), recovered complete external eye motility (“Bs” EDSS sub-score from 3 to 1), sphincter control (“Sp” EDSS sub-score from 4 to 1) and lucidity, but had significant loss of walking coordination (dynamic ataxia) as a neurological result (“Cer” EDSS sub-score from 4 to 3). At present the patient exhibits a stable EDSS of 5.5.

During SIA, no alterations in clinical chemistry were observed, apart from slight variations in fibrinogen concentration.

**Fig. 6:**
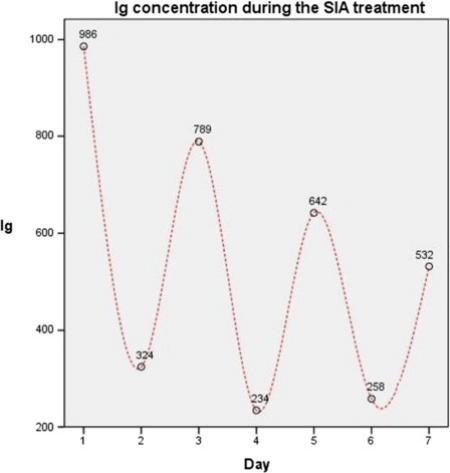
Sawtooth-like behavior of gamma globulins concentration (mg/dl) during SIA treatment

Similarly, albumin concentration was constant throughout the treatment and replacement was not necessary. On the other hand, we noted a strong reduction in total gamma globulin concentrations (IgG) immediately after the first SIA session. This modification was transient and self-limited to the 20 h following treatment, a sawtooth-like behavior caused by pulsed induction of antibody redistribution. Throughout the SIA, gamma globulins never reached one third of baseline concentrations (Fig. 6) and their level was monitored to avoid immunedepression.

We also measured all the other globulins, but no alterations were detected, apart from a slight but stable reduction in total M and A classes during SIA. In contrast, we found a significant decrease in C3 and C4 complement components throughout SIA.

The lymphocyte count and lymphocyte sub-populations were normal at all times during hospitalisation. Specifically, the CD4+/CD8+ ratio stayed between 0.8 and 1.2 during SIA and underwent no substantial modifications during the SIA-free period.

Clinically, on only one occasion did we register transient hypotension and, on another, a cold sensation with transient chills. The global perception of the patient was of minimal discomfort.

We treated the thrombophlebitis of the right femoro-popliteal axis with rapid removal of the catheter immediately after onset of fever, broad-spectrum antibiotics for 7 days and anticoagulants for 6 months. The latter was stopped after the restoration of patency of the femoro-popliteal axis after 8 months.

## Conclusions

Although steroid-refractory rebound after Fingolimod discontinuation in Multiple Sclerosis has already been described recently [18–21], its pathogenesis remains putative and is inferred from knowledge of the drug’s mechanism of action. Indeed, many authors attribute the rebound after withdrawal of Fingolimod to an immune reconstituition-like mechanism supported by increased numbers of lymphocytes in the CSF and brain, during the following 120 days after cessation of therapy. Interestingly, we instituted a treatment which is known to not interfere with the lymphocyte count, but with the humoral concentrations in pathogenetic plasma Complement and antibodies. In our study, we obtained a large reduction in these components, which was also associated with a rapid 3-point decrease in EDSS.

The successful application of PEX in several cases suggests the involvement of humoral factors [22]. Moreover, the use of selective immune adsorption columns narrows the pathogenetic players to the C3 Complement fractions and IgG [23, 24]. These observations are based exclusively on proteomic analysis of the column eluate and routine determination tests conducted on blood samples from the treated patients. The present study also showed rapid improvement of neurological condition related to falling levels of these humoral factors during SIA. In contrast, the lymphocyte sub-populations never underwent substantial alterations. Indeed, the CD4/CD8 ratio was suitable to start Natalizumab therapy.

Furthermore, due to the more selective approach concerning the pathogenetic plasma components of this procedure, the concentration of albumin remained normal during the entire course of therapy, so that the patient did not need to implement it.

From the paraclinical point of view, the expected remarkable regression of CELs was associated with whole-brain atrophy. This should be understood not as relapse-induced neurodegeneration, but rather as to the result of the anti-inflammatory effect of SIA, resulting in reduction of oedema rather than tissue components, hence SIA-induced pseudo-atrophy. Indeed, detectable disease-related brain atrophy requires at least 3 months, since it depends on slowly developing neurodegenerative mechanisms [25].

The main adverse event was the thrombophlebitis of the femoro-popliteal axis contiguous to the venous access, together with the related hyperthermia. This complication is equally common to the other intravenous procedures including PEX [26]. However, in our case, important thrombophlebitic co-factors were the urinary infection and the urinary and rectal incontinence, despite the bladder catheter and the application of all the rules of hygiene, disinfection and anticoagulation.

On the other hand, rapid clinical improvement occurred after the fourth session of SIA, as soon as the patient was afebrile.

In this regard we note that the neurological improvement was registered with respect to basal conditions, so that it was not attributed to resolution of the hyperthermia.

In summary, this article describes the case of 32-year-old relapsed MS patient undergoing a steroid-refractory rebound after Fingolimod discontinuation who experienced dramatic recovery as a result of selective serum immune adsorption (SIA). At present, although several immuno-mediated diseases benefit from the latter therapy, this is the first case of rebound in MS complication being treated with SIA to be reported in the literature. The rapid and substantial clinical improvement after treatment, with minimal side effects, gives this therapy a whole new dimension regarding the aforementioned complication in MS.

Moreover, it could be speculated that this pathological state was associated with pattern II of demyelination, taking account of the good response to treatment acting on autoantibodies and other components of immune system efferent arm. This latter effect reflects the driving force of the anti-inflammatory phenomenon responsible for the reduction of the oedema lesions which resulted in the so called SIA-induced brain pseudo-atrophy.

Thus, SIA represented an effective therapeutic strategy for this case of relapsed MS as steroid-resistent rebound post Fingolimod cessation.

## Consent

Written informed consent was obtained from the patient for publication of this Case report and any accompanying images. A copy of the written consent is available for review by the Editor of this journal.

## Abbreviations

MS: Multiple sclerosis
RRMS: Relapsing remitting multiple sclerosis
IVMP: Intravenous 6-methyl prednisolone
SIA: Selective immuno adsorption
PEX: Plasma exchange
EDSS: Expanded disability status scale
IM-TR 350: Tryptophan-immobilized polyvinyl alcohol gel column
RBON: Retrobulbar optic neuritis
CELs: Contrast enhancing lesions
MRI: Magnetic resonance imaging
TBV: Total brain volume
CSFV: Cerebrospinal fluid volume
Ig: Immunoglobulins
